# Intrinsically disordered cytoplasmic tail in netrin receptor DCC binds the large ribosomal subunit to inhibit translation

**DOI:** 10.1016/j.jbc.2025.110741

**Published:** 2025-09-18

**Authors:** Natasia Paukovich, Elizabeth A. Spear, Andrea MacFadden, Nathan N. Nowling, Morkos A. Henen, Beat Vögeli, Megan E. Filbin

**Affiliations:** 1 Department of Biochemistry & Molecular Genetics, University of Colorado Denver School of Medicine, Aurora, Colorado, USA; 2 Department of Chemistry and Biochemistry, Metropolitan State University of Denver, Denver, Colorado, USA

**Keywords:** translation, translation control, receptor structure-function, ribosome, ribosome function, neuron, neurodevelopment, intrinsically disordered protein, nuclear magnetic resonance (NMR)

## Abstract

Transmembrane receptors in neurons act as transducers of extrinsic growth cues by regulating local protein synthesis of specific mRNAs to facilitate axon guidance. In the absence of cues, receptors tether and silence translation at the membrane, but little is known about this receptor-ribosome interaction. Here, we show the direct and specific interaction between the transmembrane receptor Deleted in Colorectal Cancer, DCC, and the 60S subunit that leads to translation inhibition in the absence of DCC’s growth cue, netrin-1. We combined translation assays, equilibrium binding, and NMR spectroscopic approaches and identified the plasma membrane-proximal portion of DCC’s cytoplasmic tail, specifically residues 1123 to 1158, bind the 60S subunit. We show that this region is unstructured, providing evidence for how the subunit is tethered at the membrane. Pinpointing the electrostatic interaction between DCC and the 60S subunit protein eL5/uL18 that leads to translational silencing, we propose a two-part binding interaction that facilitates this function. Our findings reveal how DCC directly regulates local translation, shedding light on the role of transmembrane receptors in controlling protein synthesis during axon guidance.

Highly polarized cells that can be up to a meter in length, such as neurons, cannot rely solely on diffusion to deliver newly translated proteins from perinuclear ribosomes to distal cellular structures. Instead, local protein synthesis at distal regions, such as dendrites and axonal growth cones, is necessary for neuronal plasticity and synapse formation (1, 2). This process allows neurons to rapidly adjust their local proteome in response to spatial and temporal cues, leading to dendritic spine formation (3), axon steering and synaptogenesis (4, 5, 6, 7, 8), and effects such as memory consolidation (9). Activation of local translation is coupled to extrinsic cues via transmembrane receptors that directly interact with translation machinery, facilitating on-demand protein synthesis (5, 10).

The integral membrane protein Deleted in Colorectal Cancer, or DCC, identified initially as a candidate tumor suppressor (11, 12), is responsible for axon guidance toward the extracellular chemoattractant signal netrin-1, leading to differentiation into presynaptic termini (13, 14, 15). This fine-tuned response relies on DCC’s direct interaction with the ribosome (5) to locally translate a subset of mRNAs within minutes of binding netrin-1 (10). In the absence of a signal, DCC tethers silenced translation machinery to its C-terminal tail (C-tail) at the inner leaflet of the growth cone plasma membrane (5, 10). Netrin-1 binding bridges two receptors together extracellularly, triggering homodimerization by the C-tail. This is thought to release the silenced ribosomes for local translation, leading to directional growth (Fig. 1*A*) (5).

**Figure 1:**
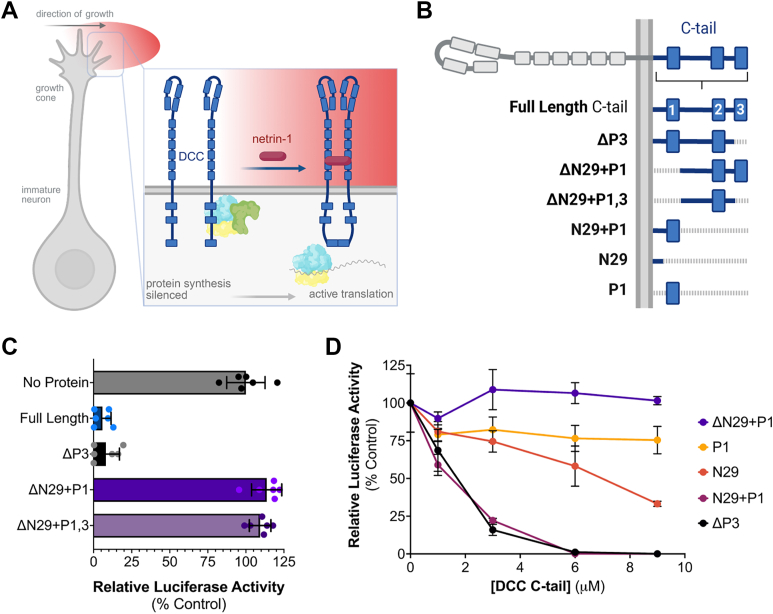
***In vitro* translation assays with DCC C-tail truncations.***A*, Schematic of how DCC and netrin-1 signaling regulate translation. In the absence of netrin-1, DCC’s C-tail binds to and stalls translation machinery at the plasma membrane. Netrin-1 induced homodimerization releases translation machinery for active translation and cell growth in the direction of the netrin-1 extracellular signal. Figure adapted from (5) with permission from Elsevier, created in BioRender. Filbin, M. (2025) https://BioRender.com/f7imzbu. *B*, C-tail truncations tested (*blue*) with conserved motifs P1-3 noted. Created in BioRender. Filbin, M. (2025) https://BioRender.com/th9f9tq. *C*, Translation efficiency (% no protein control) in the presence of 3 μM full-length C-tail, truncated proteins ΔP3 and ΔN29+P1, the combination of those two: ΔN29+P1,3. *D*, Dose-response curves of translation efficiency for membrane-proximal residues N29+P1, N29 and P1 as shown in (*B*) compared to the C-tail truncations. Data graphed as the mean of two biological replicates performed in triplicate (n = 6), with error bars ± SD.

DCC co-sediments with 40S and 60S ribosomal subunits, and the 80S ribosome, potentially engaged with an mRNA as a monosome in cells (5, 10). Yet, it does not appear to co-sediment with polysomes, implying the ribosomes associated with DCC are not actively translating (5). Indeed, while monosomes are enriched in distal regions of neurons (16) and preferentially translate mRNAs in these regions (17), the monosomes formed by DCC’s C-tail are silenced (5). Moreover, immunoprecipitation and proximity-based assays reveal DCC’s C-tail interacts with the 60S subunit protein eL5/uL18 (herein referred to as ribosomal protein L5 or rpL5) (5, 10). Upon netrin-1 signaling, this association is lost as new proteins are synthesized, suggesting ribosomes dissociate from DCC, freeing them to initiate translation (5, 10). While this cue-induced translation initiation process has been carefully examined, the way in which DCC’s C-tail silences translation in the absence of netrin-1 is not well understood.

DCC’s cytoplasmic C-tail consists of three highly conserved sequence motifs (P1-P3) separated by more variable linker sequences (18) (Fig. 1*B*). Thus far, only the N-terminal P1 motif has been shown to be important for translation regulation. P1 interacts with rpL5, and when deleted from the C-tail, netrin-1-induced translation is abrogated, implying the P1 motif is important for tethering ribosomes to DCC (5). Yet, what mediates the interaction between DCC’s C-tail and the 60S subunit, and how this complex forms so close to the plasma membrane, remains unknown.

In this study, we investigate the interaction between DCC’s C-tail and the ribosome. We discovered the minimal translation inhibitory sequence within the C-tail extends beyond the P1 motif to include the first ∼50 amino acids of the tail that extends into the cytoplasm. This part of the C-tail is entirely disordered, which we suggest facilitates its ability to tether the 60S subunit (and likely ribosome) without clashing with the membrane. The interaction with the 60S subunit is mediated by highly conserved electrostatic residues that directly interact with rpL5, and this interaction is essential for DCC-mediated translation silencing. Overall, this study provides evidence for the direct and specific interaction between the ribosome and a transmembrane receptor required for local translational control.

## Results

### Plasma membrane-proximal region of DCC’s C-tail is responsible for translation regulation

In axonal growth cones, the DCC receptor functions to promote translation in the presence of netrin-1, by releasing sequestered translation machinery from its cytoplasmic C-terminal tail (C-tail) upon netrin-induced dimerization (Fig. 1*A*) (5), raising the question: which part of the C-tail is important for regulating translation? *In vivo*, the conserved P1 and, to a lesser degree, the P3 motifs are necessary to promote translation in the presence of netrin-1 (5). However, in the absence of netrin-1, the DCC’s C-tail blocks the translation machinery from synthesizing proteins - a phenomenon mimicked *in vitro* using purified C-tail in lysate (5). To narrow down the region of DCC’s C-tail important for inhibiting translation *in vitro*, we purified a series of truncated C-tails (Fig. 1*B*) and measured the ability of these proteins to inhibit translation *in vitro*. Using a monocistronic luciferase reporter mRNA, we first measured whether the N- or C-terminus of the DCC C-tail affected translation as was shown in cells. We showed that when the P3 motif at the C-terminus of the C-tail is missing (ΔP3), translation is inhibited at levels comparable to the full-length (FL) C-tail (92% and 95%, respectively), indicating it does not play a role in stalling translation machinery at the membrane (Fig. 1*C*). These results support conclusions from *in vivo* studies that suggest the P3 motif is necessary for homodimerization that releases translation machinery upon netrin-1 signaling but not for directly tethering and stalling machinery at the membrane (5). When we removed just the N-terminus (ΔN29+P1) or both the N- and C-termini from the C-tail (ΔN29+P1,3), translation was comparable to our no-protein control. These data indicate the first 45 residues of the C-tail (N29+P1, residues 1123-1167) proximal to the plasma membrane are necessary to inhibit translation. To test whether the N29+P1 region of the C-tail is sufficient to inhibit translation *in vitro*, we measured the dose-response translation inhibition. We found the N29+P1 protein decreased translation to the same level as the ΔP3 protein over a 6-fold increase in DCC protein, while the ΔN29+P1 had no effect (Fig. 1*D*). Note that we compared N29+P1 to ΔP3 and not full-length because ΔP3 is more soluble than the full-length C-tail, and to prevent *in vitro* homodimerization that would confound the results. The N29+P1 protein contains the first 29 amino acids of the tail (those that succeed the transmembrane domain) and the conserved P1 motif, which was identified as the key translation regulatory region of DCC’s C-terminal tail *in vivo* (5). To verify whether the P1 is sufficient to inhibit translation, we measure the dose-response translation inhibition *in vitro*, using the P1 motif and the first 29 amino acids in the cytoplasmic tail (N29) individually and found that neither was sufficient to inhibit translation to the level of the N29+P1 protein (Fig. 1*D*). Importantly, we observed that the N29 protein inhibits translation to a greater degree at higher doses than the P1 protein, suggesting the residues in this region of the tail likely bind an important part of the ribosome to prevent its function.

### DCC’s translation-inhibitory region is intrinsically disordered

**Figure 2:**
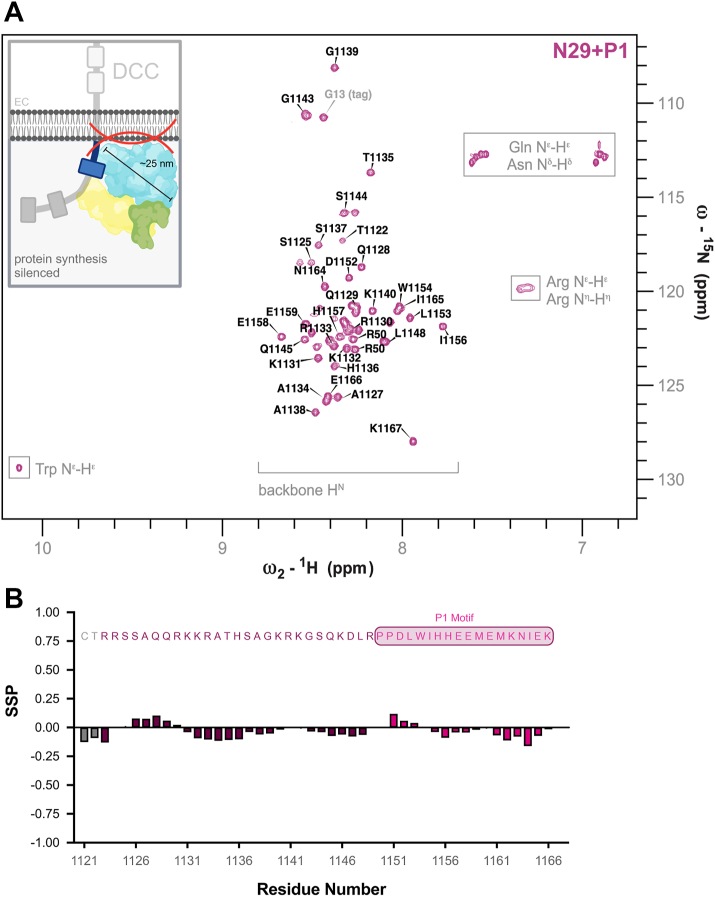
**Two-dimensional NMR of DCC C-tail N29+P1 region.***A*, Two-dimensional ^15^N-^1^H HSQC of the N29+P1 region in DCC’s C-tail, with peaks labeled per residue. Side-chain peaks boxed, and backbone amides are bracketed. Inset: Schematic of potential steric hindrance between translation machinery tethered by DCC’s C-tail N29+P1 region and the inner leaflet of the plasma membrane. Created in BioRender. Filbin M. (2025) https://BioRender.com/xmgzbyu. *B*, Secondary structure propensity (SSP) of assigned peaks corresponding to the residues in N29+P1 (shown above graph, with *grey* residues part of the transmembrane domain, and conserved P1 motif boxed). SSP values close to +1 indicate α-helical and −1 β-sheet propensity, while values near 0 indicate intrinsically disorder.

Immunogold labeling and thin-section electron microscopy show DCC and ribosomal components within 30 nm of each other and within 50 nm of the inner leaflet of the axonal growth cone plasma membrane, respectively (5, 10). The proximity of DCC’s C-tail inhibitory region to the plasma membrane poses a challenge due to steric hindrance between the membrane and translation machinery (Fig. 2*A*, inset). This is particularly true if the C-tail is folded, which would bring the stalled translation machinery even closer to the inner leaflet of the membrane. Using the DisEMBL webserver (19), we found DCC’s C-terminal tail was predicted to be disordered, particularly the N-terminal region proximal to the membrane (N29+P1, data not shown). To confirm this, we employed NMR spectroscopy. A ^15^N-^1^H heteronuclear single-quantum coherence (HSQC) experiment with ^15^N-labeled N29+P1 showed narrow dispersion of peaks in the ^1^H-dimension (Fig. 2*A*). This limited peak dispersion in the ^1^H dimension is typical of intrinsically disordered proteins (IDPs) and confirms that DCC’s N29+P1 region in the C-tail is entirely disordered.

The amino acid composition of the N29+P1 protein is not complex; it comprises many repeating amino acids, and over half of amino acids are hydrophilic, most of them charged. Despite the poor peak dispersion, the small size of N29+P1 made it possible to nearly complete amide backbone assignments for N29+P1 (Fig. 2*A*) (BMRB:51898). These assignments were performed on a doubly-labeled (^15^N, ^13^C) N29+P1 protein using a set of 3D NMR experiments. The HNCACB and CBCAcoNH experiments, which are standard for such analyses, are generally sufficient for assigning small, well-folded proteins that have well-dispersed peaks. To address challenges such as peak overlap or repeated residues, which we observed with N29+P1, we used the HNN experiment, which connects neighboring amides in a bidirectional manner to provide an additional layer of information. This approach is particularly well-suited for intrinsically disordered proteins (IDPs) since their increased backbone flexibility can overcome the generally low sensitivity generated by relatively inefficient magnetization transfer pathways to generate strong signals (20). Assignment of backbone amides was completed to 98% (excluding prolines), with R1130 as the only missing amide. Similarly, C_α_ and C_β_ atoms were assigned at 98% completeness (for non-proline residues), and 96% of carbonyl atoms were successfully assigned, excepting S1137 and P1150.

The narrow dispersion of peaks in the ^1^H dimension of the ^15^N-^1^H HSQC spectrum confirmed the disordered nature of N29+P1. However, while IDPs do not contain any stable secondary or tertiary structure, they may sample secondary structure elements in solution (21). To assess the secondary structure characteristics of N29+P1, we utilized the Secondary Structure Propensity (SSP) score (22). SSP scores for IDPs typically range between −0.2 and + 0.2, whereas fully formed β-strands score at −1.0 and fully formed α-helices at +1.0. Here, we used chemical shift data from H_N_, N, C_α_, and C_β_ atoms obtained through backbone assignments of N29+P1 to calculate SSP scores (Fig. 2*B*). While there are some trends for regions of N29+P1, all scores fall between −0.2 and + 0.2, indicating N29+P1 does not sample any secondary structure propensities. This corroborates the ^15^N-^1^H HSQC spectrum observations, leading us to conclude that N29+P1 is intrinsically disordered.

### DCC C-tail residues 1123 to 1158 region binds the free 60S subunit

**Figure 3:**
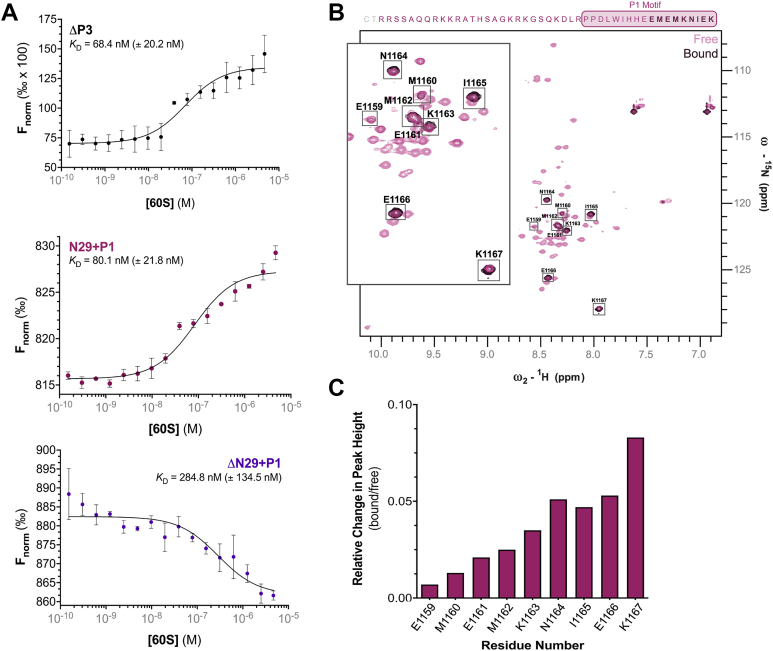
**Mapping the interaction between DCC C-tail N29+P1 and the 60S ribosomal subunit using two-dimensional NMR.***A*, Equilibrium binding assays showing normalized fluorescence (F_norm_) changes upon 60S subunit titration with DCC C-tail truncation mutants. The affinity values are noted with ΔP3 *K*_D_ = 68.4 nM (±20.2 nM), ΔN29+P1 *K*_D_ = 284.8 nM (±134.5 nM), and N29+P1 *K*_D_ = 80.1 nM (±21.8 nM). Data graphed using a single-site binding model of the mean (n = 3), with error bars ± SD and *K*_D_ ± *K*_D_ confidence (68% confidence interval). *B*, ^15^N-^1^H HSQC of N29+P1 free (transparent *magenta peaks*) overlayed with 60S (40:1 DCC:60S stoichiometry) titrated sample (*dark magenta*), with N29+P1 residues above the spectrum for reference. Peaks present in both free and bound are bolded in the reference sequence above. Peaks representing the nine most C-terminal residues (E1159-K1167 from the P1 motif) are boxed and labeled with their respective residues, as shown in the inset. *C*, Graph of the relative change in peak height (bound/free) for residues 1159 to 1167 from (*B*).

DCC’s interaction with translation machinery, including the 60S ribosomal subunit, has been shown via co-localization, -sedimentation, -precipitation and proximity ligation (within 40 nm) experiments (5, 10). Yet, whether DCC’s C-tail directly interacts with the ribosome, and what portion of the tail is responsible for this interaction, is unknown. To test this, we measured equilibrium binding with purified DCC C-tail proteins ΔP3, ΔN29+P1, and N29+P1, and 60S subunits. We found the N29+P1 protein binds to 60S subunits with a similar affinity (*K*_D_ = 80.1 nM ± 21.8 nM) as the ΔP3 protein (*K*_D_ = 68.4 nM ± 20.2 nM) - both of which also inhibit translation to the same degree. Additionally, N29+P1 and ΔP3 bind the 60S subunit with an approximately four-fold higher affinity than the ΔN29+P1 protein (*K*_D_ = 284.8 nM ± 134.5 nM), which does not inhibit translation (Figs. 1*C* and 3*A*). It should be noted that binding assays were limited by 60S subunit solubility; therefore, the *K*_D_ values are used as a measure of the relative degree of binding (not absolute measures of affinity). These data suggest that while DCC’s C-tail can broadly interact with the 60S subunit directly, the membrane-proximal N29+P1 region of DCC’s C-tail binds via a specific higher-affinity interaction that leads to translation inhibition.

Our binding studies raise the question: which residues in the N29+P1 C-tail region interact with the 60S subunit? To answer this, we took advantage of our peak assignments (from Fig. 2*A*), and the ability to measure per-residue binding interaction using NMR. At approximately 2.0 MDa, the 60S ribosomal subunit is over 50× larger than the upper detection limit of traditional solution-state NMR. Upon rigid binding to the 60S subunit, the ^15^N-labeled DCC N29+P1 protein would effectively become part of this large complex, behaving as a much larger entity. As a result, we would expect the NMR signal from N29+P1 to disappear due to the rapid relaxation rates of the larger complex, making it undetectable by NMR. Indeed, most of the resonances of N29+P1 disappeared upon addition of 60S subunit (Fig. 3*B*), supporting our equilibrium binding data (Fig. 3*A*). However, nine residues (1159–1167) at the very C-terminus of the N29+P1 protein remained detectable (Fig. 3*B*), suggesting the interaction between the N29+P1 protein and the 60S subunit is specific and does *not* involve these nine C-terminal residues. Although these residues remain detectable in the spectrum, we found their peak intensities are reduced compared to the free protein (Fig. 3*C*). This is expected as the effective tumbling time experienced by the C-terminus of the protein is decreased due to its close association with the large 60S subunit. The effect could be further enhanced by transient, non-specific interactions with the 60S subunit due to their proximity. It is likely that the decrease is exclusively due to the former, considering peak intensity of these nine residues almost continuously increases from N→C terminus (Fig. 3*C*). However, the intrinsically disordered nature of the C-terminus and its locally high tumbling rate still allow these residues to remain visible. In support of this theory, these nine C-terminal residues are in the P1 motif, which by itself only minimally decreased translation (Fig. 1*D*). Hence, these NMR data substantiate evidence that the N29 residues and only part of the P1 motif are important for binding the 60S ribosomal subunit to inhibit translation.

### Conserved electrostatic residues in DCC’s C-tail are necessary to inhibit translation

**Figure 4:**
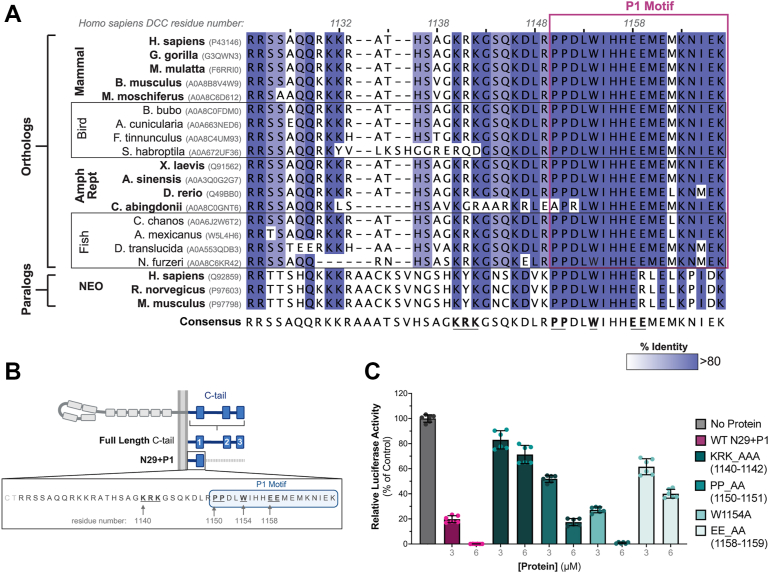
**Multiple sequence alignment to DCC C-tail N29+P1 region and *in vitro* translation assays withtargetedsubstitutions.***A*, Clustal Omega alignment of N29+P1 residues 1123 to 1167 from DCC orthologs in mammals to fish and paralogous Neogenin-1 (NEO) receptors. Shades of purple indicate conservation (>80%) with the consensus sequence shown below. Boxed in magenta are the residues corresponding to the P1 motif. Bolded and underlined residues in consensus are of interest due to sequence and chemical conservation from alignment. *B*, Schematic of residues chosen for alanine substitutions (bolded and underlined, from *A*) in the N29+P1 protein. Created in BioRender. Filbin, M. (2025) https://BioRender.com/gdm426u. *C,* Translation efficiency (% no protein control) of targeted alanine substitutions identified in *A* and shown in *B*, at 3 and 6 μM per protein. Data graphed as the mean of two biological replicates performed in triplicate (n = 6), with error bars ± SD.

DCC belongs to a subgroup within the immunoglobulin (Ig) superfamily characterized by four Ig and six fibronectin type III repeats in the extracellular domain with conserved P-motifs in the intracellular C-terminal tails (12, 23). Orthologs, from invertebrates to higher order vertebrates, and paralogs like Neogenin-1 (NEO) conserve signal transduction and function as axon guidance receptors (14, 18, 24). In fact, the evolutionary loss of the *DCC* gene in birds such as chickens (25) may be functionally compensated by the paralog NEO (26). Considering the conserved function of DCC homologs, we sought to identify key residues in DCC’s N29+P1 region, using sequence conservation. We supposed that highly conserved residues in this region may form important interactions with the 60S subunit that leads to translation inhibition. Multiple sequence alignment of DCC orthologs from fish to mammals and mammalian neogenin paralogous sequences reveal variable sequence identity amongst residues in the N29 region and greater than 80% identity for most of the P1 motif (Fig. 4*A*). The consensus sequence from the alignment is nearly identical to the human DCC protein, with four additional residues due to the slightly longer neogenin sequence. Using this information, we took a targeted approach focused on the highly conserved residues toward the middle of the N29+P1 region (between residues 1140 and 1159), which is the region we identified important for translation inhibition and 60S subunit interaction (Figs. 1*D* and 3). Importantly, we propose residues 1123 to 1138 are likely too close to the inner leaflet of the plasma membrane to bind the large 60S subunit (see Fig. 3*A*, inset), and we show that residues 1160 to 1167 do not interact with the subunit (Fig. 3*B*). Within our target region, we substituted alanines for: (1) basic residues at 1140 to 1142 (KRK→AAA), (2) two proline residues 1150 to 1151 (PP→AA), (3) tryptophan 1154 (W1154A), and (4) acidic glutamates 1158 to 1159 (EE→AA) to disrupt electrostatic and structural contributions to function (Fig. 4*B*). We tested the ability of these alanine-substituted N29+P1 proteins to inhibit *in vitro* translation of a monocistronic luciferase reporter mRNA at two different concentrations and found that disrupting the basic and acidic residues affected the ability of the N29+P1 protein to inhibit translation more than the other substitutions (Fig. 4*C*). In fact, translation in the presence of the KRK→AAA protein only decreased by approximately 20% percent, compared to the approximately 90% decrease by WT N29+P1. Collectively, our translation results indicate both the N29 and the P1 regions are necessary to inhibit translation *in vitro* (Fig. 1*D*), likely due to electrostatic interactions primarily driven by the basic residues at 1140 to 1142 (in the N29 region), and partially by the acidic residues at 1158 to 1159 (in the P1 region).

### DCC’s C-tail binds the 60S subunit via electrostatic interactions with eukaryotic ribosomal protein L5 (uL18)

**Figure 5:**
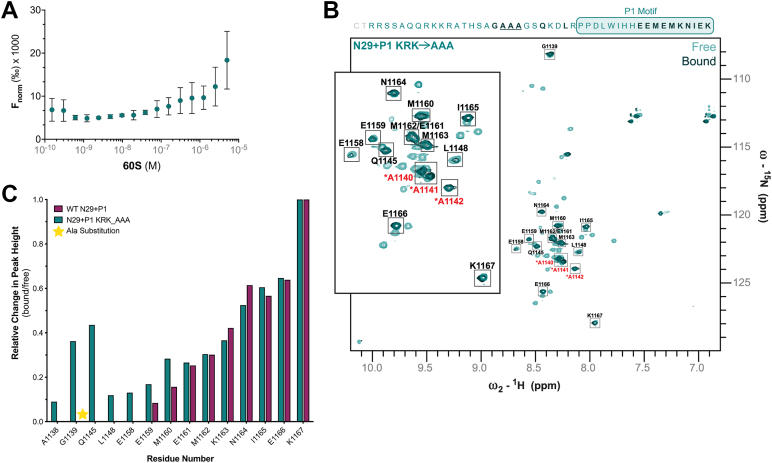
**Alanine substituted N29+P1 interaction with 60S subunit via two-dimensional NMR mapping.***A*, Equilibrium binding assays showing normalized fluorescence (F_norm_) changes upon 60S subunit titration with N29+P1 KRK→AAA protein. The mean (n = 3), with error bars ± SD are graphed with the exception of the highest titrant (5 μM 60S subunit) which is n = 2. *B*, ^15^N-^1^H HSQC of KRK→AAA N29+P1 free (transparent *teal peaks*) overlayed with 60S (40:1 DCC:60S stoichiometry) titrated sample (*dark teal*), with KRK→AAA N29+P1 residues above spectrum for reference (substituted alanines underlined). Peaks present in both free and bound are bolded in the reference sequence above and boxed and labeled with their respective residues in the spectrum. Inset shows most peaks that remain unchanged in free and bound states, including the 10 most C-terminal residues (E1158-K1167 from the P1 motif). Residues A1140-A1142 (mutated alanines) have not been assigned and are labeled (*red*) arbitrarily to the three new peaks. *C*, Graph of the relative change in peak height (bound/free) for WT N29+P1 (*magenta*) and KRK→AAA N29+P1 (*teal*) residues 1138 to 1167 upon 60S addition. Intensity changes are normalized. *Yellow* star represents the site of alanine substitutions.

Our functional data suggest electrostatics drive DCC’s interaction with the translation machinery. In particular, the patch of basic residues between 1140 to 1142 seem to make important interactions that lead to decreased translation. To identify whether these residues drive contacts with the 60S subunit, we used the same equilibrium binding and NMR methods as above (Fig. 3) to assess changes in 60S subunit binding with the KRK→AAA protein compared to WT N29+P1. We found the KRK→AAA protein has a substantial decrease in relative affinity for the 60S subunit; no binding was observed in the lower nanomolar range where we observed WT N29+P1 binding (*K*_D_ = 80.1 nM ± 21.8 nM), and we were unable to fit a binding isotherm to the experimental data in the same 60S subunit concentration range as we measured in Figure 3. At the highest concentrations of the 60S subunit (2.5–5 μM) we did observe an increase in normalized fluorescence (Fig. 5*A*), indicating specific interaction between the KRK→AAA protein and the 60S subunit. We therefore predict the KRK→AAA protein binds the 60S subunit in the lower micromolar range, representing at least a 10-fold decrease in affinity for the 60S subunit compared to WT N29+P1. To pinpoint which residues lose interaction, we compared the ^15^N-^1^H HSQC spectrum of KRK→AAA free and bound to 60S subunit. The same nine C-terminal residues (1159–1167) we observed in the WT N29+P1-60S spectrum, are present in the KRK→AAA-60S spectrum, indicating these residues are not interacting with the 60S (Fig. 5*B*). Additionally, we observed more residues (or rather, residues that stayed detectable in the bound form), including the substituted alanine residues (1140 to 1142), as well as A1138 and G1139 which are immediately N-terminal of the alanines, all of which do not interact with the 60S. These data are consistent with the observed decreased affinity (Fig. 5*A*) and with our hypothesis that electrostatic interactions, which previously held those residues close to the 60S subunit, have been disrupted. When we compare the relative change in peak height (bound/free) between the KRK→AAA and WT N29+P1 proteins, we observe a broader loss of interaction between the alanine-substituted protein and 60S subunit, particularly in the N29 region (1123–1149), compared to the WT N29+P1 interaction (Fig. 5*C*). Interestingly, one of these residues, Q1145, retains significant peak height compared to the other visible peaks in this area, indicating it has more conformational freedom or local flexibility than the others. We found that this particular residue, along with G1139, also exhibit large chemical shift perturbations (CSPs) in the unbound state (Fig. S1), indicating the alanine substitutions alter the chemical environment both locally and distally, likely leading to the observed broad effect on 60S subunit binding. Lastly, we observed that the peak of residue E1158 in the P1 motif was not present in the WT N29+P1-60S spectrum, and a more prominent peak for E1159 (Fig. 5*C*), indicating an additional loss of electrostatic interaction between the KRK→AAA protein and 60S subunit. While we show these glutamate residues in the conserved P1 motif contribute to translation inhibition (Fig. 4*C*), these data support the larger effect on translation for the KRK→AAA protein as it more broadly affects 60S subunit interaction.

**Figure 6:**
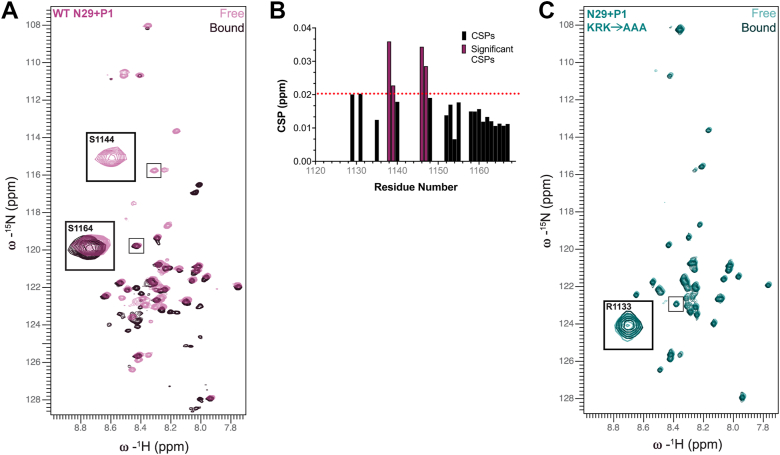
**Mapping the interaction between 60S subunit ribosomal protein eL5/uL18(rpL5)and the N29+P1 protein via two-dimensional NMR.***A*, ^15^N-^1^H HSQC of WT N29+P1, showing free spectrum (*light magenta*) overlayed with bound (to rpL5) spectrum (*dark magenta*). *Gray* boxes highlight examples of peaks not shifting (S1164) and shifting (S1144) whether or not they reappeared elsewhere in the spectrum. *B*, Chemical shift perturbation (CSP) plot between free and bound of WT N29+P1 by residue. *Black bars* indicate CSPs that are not significant, while *magenta bars* indicate significant shifts. Significance was calculated as average + 1 standard deviation. Any residues which do not have an associated bar indicate residues which have disappeared upon addition of rpL5 (whether or not they reappeared elsewhere in the spectrum). *C*, ^15^N-^1^H HSQC of free KRK→AAA N29+P1 spectrum (*light teal*) overlayed with bound spectrum (to rpL5) (*dark teal*). The gray box highlights example of residue (R1133) not shifting.

The conserved P1 motif in DCC’s C-tail interacts with ribosomal protein eL5/uL18 (referred to as rpL5) (5), a component of the 60S subunit central protuberance that forms an intersubunit bridge with the 40S subunit (27). Given our NMR data showing the KRK→AAA broadly disrupts interaction with the 60S subunit (including with the P1 motif), we hypothesized that this may be due to a loss of interaction with rpL5. To test this, we first measured whether the N29+P1 protein interacts with purified rpL5, and then whether interaction was ablated due to the alanine substitutions in the KRK→AAA protein using two-dimensional NMR. The ^15^N-^1^H HSQC spectrum of N29+P1 and rpL5 (at a 1:1 M ratio) show significant peak shift and disappearance (Fig. 6*A*). We quantified and graphed peak shifts/disappearances and show that the residues corresponding to the nine residues in the P1 motif that do not interact with the 60S subunit (Fig. 3*B*), maintained identifiable peaks, all with CSPs below average, in the bound state (Fig. 6*B*), indicating these residues do not interact with rpL5. On the other hand, we noticed the KRK residues at 1140 to 1142 disappear in the presence of rpL5, and flanking residues exhibited the largest CSPs, indicating these residues are binding rpL5. It should be noted that we did not assign peaks that appeared in the bound (+rpL5) spectrum compared to the free protein spectrum, and that we take these disappearances/shifts merely as indicators of direct interaction between DCC’s C-tail and rpL5. When we measured the interaction between the KRK→AAA N29+P1 protein and rpL5, we observed no visible CSPs or disappearances when comparing the free to bound (+rpL5) samples (Fig. 6*C*), indicating the alanine substitutions preclude DCC-rpL5 binding. These data suggest that the interaction between DCC’s C-tail and rpL5 are driven by the KRK residues (1140–1142) directly, but this interaction alone does not mediate 60S subunit tethering by DCC’s C-tail (Fig. 5*B*).

## Discussion

We provide evidence for direct and specific interaction between DCC’s C-tail and the 60S subunit that leads to translation inhibition. We show that this interaction, and the resulting translation inhibition, is mediated by electrostatics between the unstructured membrane-proximal DCC C-tail and principally ribosomal protein L5 within the 60S subunit.

Based on the structural data in this study, we propose that tethering of the 60S, and by extension ribosomes, in cells, requires unstructured and flexible C-tails of receptors to mediate this process. While there is evidence of ribosomes directly associated with membranes, they are either translating proteins into the endoplasmic reticulum (28), or they are hibernating ribosomes on the mitochondrial membrane, stalled under nutrient deprivation (29). In the case of DCC, the tethered ribosomes are only membrane-proximal until netrin-1 signaling induces their release for local protein synthesis (10). Yet, electron micrographs reveal that the DCC-bound ribosomes are within 50 nm of the membrane (5, 10), raising the question: how do these tethered ribosomes avoid steric clashing with the inner leaflet of the neuronal membrane? This is particularly significant considering the portion of the DCC that binds the ribosome includes the first ∼45 residues (N29+P1) in the cytoplasmic tail. We found that this region of the C-tail is completely disordered, making the protein backbone flexible and extended. If we approximate the C_α_-C_α_ length at 0.4 nm (30), the estimated length of this portion of the C-tail would be ∼18 nm. The human 60S ribosomal subunit has a diameter of approximately 25 nm, and a hydrodynamic radius of approximately 15.0 nm in cells (31), making it possible to bind the membrane-proximal region of DCC’s C-tail without membrane interference. *In situ* cryogenic electron tomography studies with subtomogram averaging of ribosomes tethered to DCC at the plasma membrane would provide high-resolution (sub-5 Å) information to resolve spatial constraints at the membrane and support our prediction.

The 60S subunit has a large binding footprint with DCC’s cytoplasmic tail, including part of the conserved P1 motif, supporting evidence that this region is necessary for translation in cells (5). However, we also show that residues N-terminal (closer to the membrane) are important for 60S subunit interaction, including a highly conserved three-residue basic patch that mediates interaction with 60S subunit protein L5. Interestingly, the interaction between DCC and rpL5 is necessary to inhibit protein synthesis, but not necessary for DCC-60S subunit complex formation. This suggests that it is not merely DCC-60S subunit interaction (or ribosome sequestration at the membrane) that leads to translation silencing (in the absence of netrin-1), but that there is a specific ribosome-inhibitory mechanism initiated by DCC. We propose that the larger binding footprint helps to coordinate the interaction between DCC and rpL5, which leads to either stalling monosomes on mRNA (10) or preventing the 60S subunit from engaging a preinitiation complex (mRNA engaged with a 40S subunit complex, (5)).

Ribosomal protein L5 is a key component of the 60S subunit, forming a region called the central protuberance along with the 5S rRNA and ribosomal protein eL11/uL5. The protuberance facilitates an intersubunit bridge (B1b/c) with the 40S subunit (27, 32), and is centralized in the ribosome to connect the peptidyltransferase and decoding centers, as well as influence elongation factor binding (as reviewed in (33)). In bacteria and yeast, this intersubunit bridge facilitates initiation and maintains translation fidelity (34, 35). It therefore seems probable that DCC’s interaction with rpL5 affects the intersubunit bridge formation or function, abrogating translation initiation. Indeed, there is evidence for unstructured proteins binding the central protuberance at the B1b/c bridge to regulate translation. For instance, the yeast protein Stm1 fastens the subunits together across this bridge to silence translation during nutrient deprivation (27, 36). Incorporating crosslinking mass spectrometry, x-ray crystallography or single-particle cryogenic electron microscopy would provide high-resolution evidence of the location and probable interactions between DCC’s C-tail, rpL5 and the 60S subunit, and support our theory that DCC’s C-tail inhibition mechanism involves this region of the ribosome.

Our data, along with previous studies about receptor regulation of protein synthesis (5, 10), converge on a broad mechanism for spatiotemporally regulating translation. The chemotropic receptors Neuropilin-1 and Robo2 interact with translation machinery to facilitate local translation (10). The C-tail of Robo2 in particular has homology with DCC’s C-tail inhibitory region (N29+P1) and is predicted to be highly disordered (data not shown). Additionally, the C-tail of DCC’s paralog Neogenin-1, which is thought to functionally replace DCC in bird lineages where the *DCC* gene is lost (25, 26) is also predicted to be highly disordered (data not shown). We therefore suggest that these receptors tether translation machinery near the membrane, facilitated by their unstructured and flexible C-tails, and prevent ribosomes from initiating protein synthesis by directly contacting functionally essential regions of the ribosome, such as the 60S subunit central protuberance and/or intersubunit bridges.

In conclusion, our findings describe the minimal region necessary for translation inhibition by DCC’s cytoplasmic tail and begin to unravel the specific interactions allowing for this ribosomal regulation. This work lends insight into the biochemical mechanisms behind cue-mediated translation and provides a framework for further study of this mechanism for translation regulation by other receptors.

## Experimental procedures

### Generating plasmids

Firefly luciferase reporter construct containing the *H. sapiens* beta-globin mRNA 5′ untranslated region (nt 1–50) in the pDBS vector was a kind gift from J.S. Kieft (37). Truncations from *H. sapiens* DCC C-terminal cytoplasmic tail ((22)) were cloned into expression plasmids as shown in Table 1. The two terminal residues of the transmembrane domain (1121–1122) were included in the truncations to provide a terminal cysteine for labeling. The N29 protein was synthesized by the T. Curtius Peptide Core at the University of Colorado Boulder, and the P1 protein was synthesized by GenScript. Alanine substitutions at residues 1140 to 1142 (KRK_AAA), 1150 to 1151 (PP_AA), 1154 (W_A), and 1158 to 1159 (EE_AA) in the N29+P1 were introduced to the pET-28a(+) vector using Q5 site-directed mutagenesis (NEB). Ribosomal protein eL5/uL18 (*H. sapiens* rpL5, NP_000960.2) was cloned into a pET-28a(+) plasmid from a gene block (IDT) using NcoI/BamHI.

**Table 1:** DCC truncation proteins & expression plasmids

Protein	Residues	Plasmid	Restriction sites
FL	1121–1447	pET-28a(+)	NdeI/BamHI
ΔP3	1121–1424	pET-15b	NdeI/XhoI
ΔN29+P1	1168–1447	pET-15b	NdeI/XhoI
ΔN29+P1,3	1168–1424	pET-15b	NdeI/XhoI
N29+P1	1121–1167	pET-28a(+)	NdeI/BamHI
N29^a^	1121–1149	N/A	N/A
P1^a^	1150–1167	N/A	N/A

FL, full-length C-tail.

a Proteins chemically synthesized.

### DCC C-tail recombinant protein expression & purification

All proteins except the N29 and P1 proteins were recombinantly expressed in *E. coli* LOBSTR-BL21(DE3) at 37 °C for 4 h. All proteins contained a N-terminal 6× His-tag and were first IMAC-purified using gravity flow. Cells were lysed (20 mM Tris pH 7.5, 500 mM NaCl, 2 mM β-mercaptoethanol, 10% glycerol, 20 mM imidazole, protease inhibitor) and the clarified supernatant was incubated with equilibrated Ni-NTA beads (Thermo Fisher) for 1 h at 4 °C. Beads were then washed (20 mM Tris pH 7.5, 500 mM NaCl, 2 mM β-mercaptoethanol, 5% glycerol, 40 mM imidazole) and eluted (20 mM Tris pH 7.5, 150 mM NaCl, 2 mM β-mercaptoethanol, 5% glycerol, 200 mM imidazole) at 4 °C. IMAC elution fractions were then concentrated in Amicon Ultra centrifugal filters (Millipore-Sigma) and further purified using Superdex 75 gel filtration chromatography in 20 mM Tris pH 7.5, 150 mM NaCl, 2 mM β-mercaptoethanol, 5% glycerol.

^15^N/^13^C-labeled N29+P1 protein was expressed in *E. coli* LOBSTR-BL21(DE3) in M9 media at 20 °C for 16 h and IMAC-purified as described above, followed by Superdex 75 gel filtration chromatography in 200 mM Phosphate Buffer pH 6.4, 100 mM NaCl, 2 mM β-mercaptoethanol. ^15^N-labeled N29+P1 protein for 60S binding was purified in 100 mM Phosphate Buffer pH 6.4, 50 mM NaCl, 2 mM MgCl_2_, 2 mM β-mercaptoethanol.

Protein purity was verified using SDS-PAGE and Coomassie staining, and concentrations were determined using molar extinction coefficients and NanoDrop A_280_ readings. The N29 protein does not contain any Trp or Tyr residues; therefore, A_205_ was used (38).

### Ribosomal subunit purification

Rabbit 60S subunits were purified from rabbit reticulocyte lysate (Green Hectares). Lysate with protease inhibitor, RNase inhibitor, and 0.2 mM DTT was gently clarified at 20,000*g* for 30 min, and the supernatant was further clarified at 40,000 rpm in a 50.2 Ti rotor for 3.5 h at 4 °C. The glassy pellet containing ribosomes was gently resuspended in 20 mM Tris-HCl, pH 7.5, 50 mM KCl, 4 mM MgCl_2_, and 2 mM DTT. Ribosomes were split in the presence of 1 mM puromycin on ice for 10 min and at 37 °C for an additional 10 min. Initiation factors were removed using 4 M KCl added to a final concentration of 500 mM in a dropwise manner. Subunits were then separated over a 15 to 30% sucrose gradient in 20 mM Tris-HCl, pH 7.5, 3 mM MgCl2, 0.5 M KCl, 2 mM DTT at 19,300 rpm in a SW28 rotor for 17 h at 4 °C. The gradients were fractionated using a piston fractionator (BioComp Instruments Ltd), and subunits were concentrated using Amicon Ultra centrifugal filters (Millipore-Sigma) and buffer exchanged into 20 mM Tris-HCl pH 7.5, 100 mM KCl, 2 mM MgCl_2_, 1 mM DTT, 0.1 mM EDTA, 0.25 M sucrose for storage. The 60S subunits used for NMR were buffer exchanged into 100 mM Phosphate Buffer pH 6.4, 50 mM NaCl, 2 mM MgCl_2_, 2 mM β-mercaptoethanol.

Subunits were quantified using a NanoDrop (Thermo Fisher) with 1 A_260_ = 29 pmol/ml. Subunit purity was verified by extracting rRNA using TRIzol (Invitrogen) followed by formamide denaturing agarose gel electrophoresis as well as transmission electron microscopy of 100 nM subunit stained with 2% (w/v) uranyl acetate on carbon 200 mesh copper grids (EMS).

### Protein labeling and binding assays

Pure ΔP3, ΔN29+P1, and N29+P1 proteins were Cy5 labeled using Monolith Protein Labeling Kit RED-maleimide chemistry (NanoTemper Technologies) by mixing equal volumes 2 μM protein (in 10-M excess TCEP) with 6 μM dye (in 100% anhydrous DMSO). The reaction was incubated for 45 min at room temperature, then dialyzed overnight in 20 mM Tris-HCl pH 7.5, 100 mM KCl, 2 mM Mg(OAc)_2_, 1 mM DTT, 0.5 mM EDTA, 0.25 M sucrose at 4 °C. Proteins were then concentrated and washed using Amicon Ultra centrifugal filters.

Microscale thermophoresis (NanoTemper Technologies) was used to measure binding affinities, using the Pico RED detector with labeled DCC proteins (target) at 5 nM and purified 60S subunits (ligand) from five uM to 0.15 nM (over 16 capillaries) in 20 mM Tris-HCl pH 7.5, 100 mM KCl, 2 mM Mg(OAc)_2_, 1 mM DTT, 0.5 mM EDTA, 0.25 M sucrose. Measurements were conducted using 20% LED excitation power, 40% (medium) IR laser power with an on-time of 2.5 s at 25 °C. Fluorescence data were normalized and fitted in NanoTemper MO. Affinity Analysis using 1:1 binding model with *K*_D_ values reported ± *K*_D_ confidence (68% confidence interval) (v.2.3). GraphPad Prism (v.10) was used for error analysis and graphing the nonlinear regression. Microscale thermophoresis was used to measure the *K*_D_ for N29+P1 and ΔN29+P1, while initial fluorescence was used for ΔP3.

### *In vitro* transcription

Monocistronic DNA, with the beta-globin 5′ UTR and firefly luciferase ORF, for *in vitro* transcription was generated by PCR with *Pfu* polymerase using a 5′ T7_BetaGlobin Forward primer (TAATACGACTCACTATAGGACATTTGCTTCTGACATAGTTGTG) and Firefly_Luc Reverse primer (GAATTACACGGCGATCTTTCC). Amplicons were purified using the Wizard DNA Extraction kit (Promega) and 1 μg of DNA was transcribed and capped in a 20 μl reaction using HiScribe T7 ARCA mRNA Kit (NEB) by incubating at 37 °C for 30 min according to the manufacturer's protocol. DNA was digested with 2 μl DNase I at 37 °C for 15 min. RNA was purified using TRIzol (Invitrogen) followed by a chloroform extraction and room-temperature precipitation using isopropanol. RNA was resuspended in nuclease-free water and precipitated again using NH_4_OAc and 100% v/v ethanol at −80 °C, followed by a final wash with 75% ethanol. RNA quantity, purity and quality were assessed using NanoDrop spectroscopy and urea-denaturing PAGE with ethidium bromide staining.

### *In vitro* translation assays

Nuclease-treated rabbit reticulocyte lysate (RRL, Promega) and ARCA-capped firefly luciferase mRNA were used for *in vitro* translation reactions. Reactions were in 10 μl containing 4 μl RRL, 0.2 μM amino acids, 0.5 μl RNasin (Promega), 1 ng RNA and the indicated protein concentrations diluted in 20 mM Tris-HCl pH 7.5, 150 mM NaCl, 2 mM β-mercaptoethanol, 5% glycerol. Reactions were incubated at 30 °C for 1 hour and halted by adding 45 μl ice-cold 1X passive lysis buffer (Promega) and cooling on ice. Luciferase activity was immediately measured using Luciferase Assay Reagent (Promega) and a Glomax Navigator Microplate Luminometer (Promega) via the manufacturer’s Luciferase Assay System program. Relative light unit data was processed in Microsoft Excel such that the No-DCC protein control was set to 100% Relative Luciferase Activity and all other reactions as percentages of the control. Data was graphed in GraphPad Prism (v.10).

### Multiple sequence alignment

Orthologous protein sequences were identified using *H. sapiens* DCC C-tail residues 1123 to 1447 (P43146) and protein BLAST (blastp) through UniProt from UniProtKB reference proteomes and the Swiss-Prot database. Alignments were based on the BLOSUM45 matrix with the E-threshold set at 100 to identify distant orthologs. Orthologs were cross-referenced and grouped using the OrthoDB (v.11) catalog (39). Only UniProt entries with complete receptor sequences were aligned, and four orthologs per group were chosen (highest and lowest percent identity with E-values ranging from 1e-18 to 7e-07). Paralogous sequences were identified using gene HGNC:2701 in the Ensemble webserver (Release 112, May 2024), followed by blastp to narrow down paralogs that align to DCC C-tail residues 1123 to 1447. All sequences were aligned using Clustal Omega (40) and visualized in JalView (41).

### NMR

^13^C- and ^15^N-labeled DCC N29-P1 was prepared in 200 mM Na-Phosphate Buffer, pH 6.4, 100 mM NaCl, and 2 mM DTT at a concentration of 125 μM. Backbone assignment was achieved by measuring ^15^N-^1^H HSQC, 3D HNCACB, and CBCAcoNH experiments on a triple-resonance Varian 900 NMR cryoprobe spectrometer at 25 °C in a 5-mm Shigemi tube. Additionally, the HNN experiment was recorded on BRUKER 600 with a cryoprobe in a 3 mm tube. For the 2D ^15^N-^1^H HSQC, 64 points were measured in the indirect dimension. Non-uniform sampling (NUS) was used for the 3D experiments with sampling schemes generated by NUS@HMS software (42). For the CBCAcoNH/HNCACB experiments, 25/30% of 96 and 80 complex points in the ^15^N and ^13^C dimensions, respectively, were sampled. For the HNN experiment, 30% of 50 and 128 complex points in the two indirect ^15^N dimensions were sampled. 2048 complex points were collected in the direct dimension. The number of scans was set to 32 for the HSQC, with interscan delays of 1.2 s. The number of scans was set to 24 for the HNCACB and 16 for CBCAcoNH and HNN, with interscan delays of 1.0 s. For the HSQC, spectral widths were set to 6.7 ppm and 27.8 ppm for ^1^H and ^15^N, respectively. Spectral widths for the HNN experiment were 13.7 ppm and 34.9 ppm for ^1^H for ^15^N, respectively. For HNCACB and CBCAcoNH experiments, spectral widths were 15.6 ppm for ^1^H, 35.5 ppm for ^15^N, and 70.4 ppm and 80.4 ppm for ^13^C, respectively. To reconstruct the 3D NUS spectra, we used the hmsIST software (42).

The binding between labeled DCC and ribosomal 60S and rpL5 was measured using ^15^N-^1^H HSQC on the BRUKER 600 with cryoprobe at 25 °C. For the DCC-60S subunit measurements: 50 μM of ^15^N WT N29+P1 or 37 μM of KRK→AAA N29+P1, with or without the addition of 1.5 μM unlabeled ribosomal 60S subunit, was in the phosphate buffer stated in the above paragraph. Experiments were measured with 100 points in the indirect dimension with 150 scans to enhance the S/N. There was a 1.3 s interscan delay. The spectral widths were 34.9 ppm and 13.7 ppm for ^15^N and ^1^H, respectively. For the DCC-rpL5 measurements, 35 μM ^15^N WT N29+P1 or 80 μM KRK→AAA N29+P1 was titrated with a 1:1 ratio of unlabeled rpL5 in the phosphate buffer stated in the above paragraph, supplemented with 5% v/v glycerol. Experiments were measured with 200 points in the indirect dimension with 64 scans. There was a 1.3-s interscan delay. The spectral widths were 34.9 ppm and 13.7 ppm for ^15^N and ^1^H, respectively.

Data processing was completed with NMRPipe and NMRDraw software (43). Visualization, resonance assignment, and CSP analysis were completed with CcpNMR V2.5.2 software (43, 44).

### Chemical shift analysis

DCC N29+P1 chemical shifts from the assignment spectra, HN, N, Cα, and Cβ, were analyzed to discern any regions containing secondary structure. Secondary Structure Propensity score (SSP) (22), ranges from −1.0, indicating a fully formed β-strand to +1.0, indicating fully formed α-helical structure. Disordered regions typically hover around 0.

Changes in peak intensity (chemical shift perturbation: CSP) between free WT N29+P1and KRK→AAA N29+P1 as well as bound to the 60S ribosomal subunit were determined via peak height in the ^15^N-^1^H HSQC experiments. The difference in peak height (Δ height) was calculated between samples, and this value was plotted. Significance was determined as CSP greater than average + half standard deviation. Only peaks for residues that appeared in both compared spectra were analyzed.

## Data availability

The NMR spectral and quantitative data for DCC N29+P1 protein generated during this study have been deposited in the Biological Magnetic Resonance Data Bank (BMRB) and are accessible under the accession code BMRB ID: 51898 (https://bmrb.io/).

## Supporting information

This article contains supporting information.

## Conflict of interest

The authors declare that they do not have any conflicts of interest with the content of this article.
