# Supplementary material for: Intrinsically disordered cytoplasmic tail in netrin receptor DCC binds the large ribosomal subunit to inhibit translation
Source: J Biol Chem. 2025 Sep 18;301(11):110741. doi: 10.1016/j.jbc.2025.110741 (PMC12556795; doi:10.1016/j.jbc.2025.110741)
Supplement: Supporting information [file mmc1.pdf]

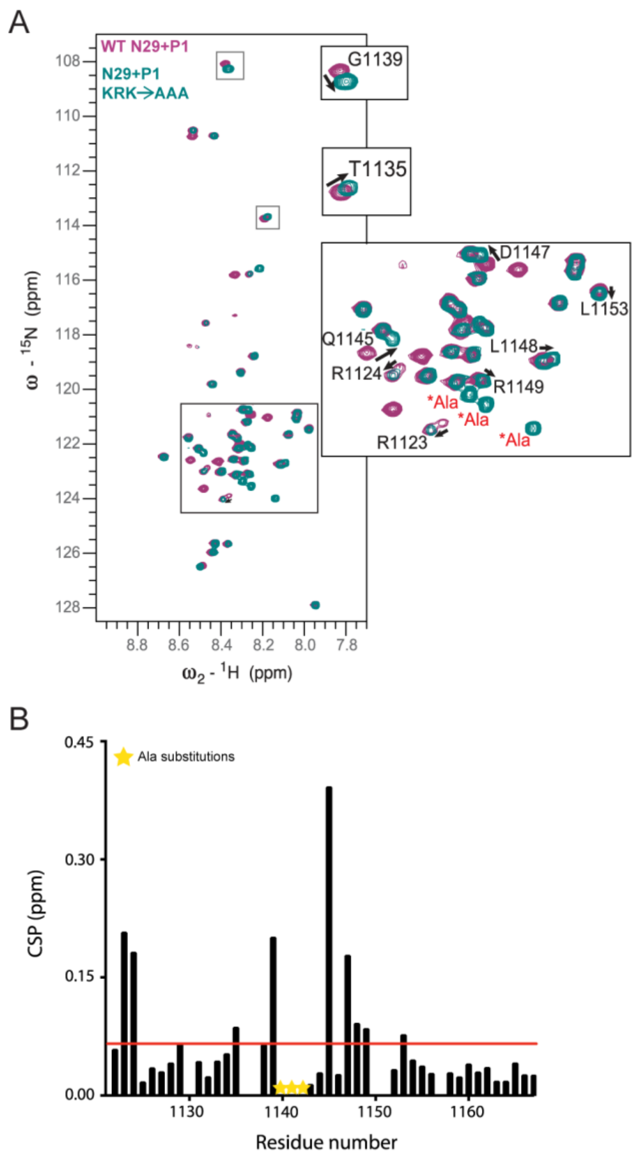

Figure S1: Two-dimensional NMR comparison of WT and KRK→AAA N29+P1 proteins. A. ^{15}N-^{1}H HSQC of WT N29+P1 (*magenta peaks*) overlaid with KRK→AAA N29+P1 mutant (*teal peaks*). Inset: newly appeared alanine peaks are arbitrarily labeled (*red*). Shifting peaks are denoted with arrows, including peaks near the site of mutation and distal from this site. **B.** Chemical shift perturbation (CSP) between WT N29+P1 protein and the KRK→AAA mutant. Depicted here are only the residues of the N29+P1 peptide and do not include any N-terminal purification tags, which may have exhibited shifts. The red horizontal line represents the average CSP + 1 standard deviation. Yellow stars represent site of alanine substitutions.
